# Benefits of Sea Cucumber Viscera on Gut Microbiota and Their Implications for Health

**DOI:** 10.3390/biology15040365

**Published:** 2026-02-22

**Authors:** Hao Zhong, Huange Zhang, Weiming Liu, Muhammad Hussain, Hui Chen, Pengbo Cui

**Affiliations:** 1 College of Food Science and Technology, Zhejiang University of Technology, Hangzhou 310014, China; 2 Zhejiang Biosan Biotech Co., Ltd., Hangshui 323000, China

**Keywords:** sea cucumber viscera, gut microbiota, bioactive compounds, by-product valorization, metabolic health

## Abstract

Sea cucumber viscera, the primary by-product of processing, are generated in increasing quantities annually, leading to significant environmental pollution and resource wastage. Nevertheless, they are rich in nutrients and possess diverse bioactivities. This review provides a detailed elucidation of the bioactive components found in sea cucumber viscera, such as peptides, sulfated polysaccharides, saponins, and lipids. It further elaborates on how these visceral bioactives reshape the gut microbial ecosystem—notably by increasing the abundance of beneficial genera, such as *Akkermansia*, *Lactobacillus*, and *Bifidobacterium*, and promoting the production of beneficial metabolites such as short-chain fatty acids and indole derivatives. Through these mechanisms, the components demonstrate potential, either directly or indirectly, to improve glucose and lipid metabolic disorders, enhance immune function, alleviate inflammation-related diseases, exert anti-aging effects, and reduce uric acid levels. However, the molecular mechanisms through which individually extracted bioactive components from sea cucumber viscera exert their health benefits by modulating the gut microecology in animal models or clinical trials remain to be fully elucidated. This review aims to offer a scientific foundation for the high-value application of sea cucumber viscera.

## Simple Summary

Sea cucumber processing generates substantial waste from internal organs (viscera), posing an environmental concern. This review highlights that these discarded viscera are a valuable source of health-promoting compounds. We explain how substances from the viscera can improve human health by supporting beneficial gut bacteria. Increasing these good bacteria helps the body better regulate blood sugar and cholesterol, boosts immunity, reduces inflammation, slows aging-related damage, and lowers high uric acid levels. Our work demonstrates that sea cucumber viscera should be repurposed, not discarded. Using them to develop ingredients for functional foods or supplements offers a sustainable way to turn waste into a resource for improving health.

## 1. Introduction

Sea cucumbers (*Holothuroidea*) are marine organisms that have long been valued in many cultures, particularly in China, for their high nutritional and medicinal properties. The body of sea cucumber comprises a thick leather-like body wall, which is the edible component and has been the major focus of bioactive compound research. The growing scale of sea cucumber farming and processing has led to significant by-product generation. Notably, the viscera—comprising the intestine, gonads (including eggs and sperm), respiratory trees, Cuvierian tubules, Polian vesicles, and associated structures—can account for up to 50% of the organism’s biomass [1,2,3]. Disposal of these viscera not only poses environmental concerns but also represents a substantial waste of nutrient-rich material [4]. Studies indicated that sea cucumber viscera are rich in proteins, polysaccharides, saponins, fatty acids, and other bioactive constituents, with a compositional profile similar to that of the body wall [5,6,7]. In vivo studies indicate that supplementation with sea cucumber visceral extracts can alter gut microbiota and demonstrate several potential health advantages, including anti-inflammatory, antioxidant, glucose- and lipid-regulatory, immunomodulatory, and anti-fatigue properties [8,9].

The gut microbiota, an intricate community primarily consisting of bacteria from the phyla *Firmicutes* and *Bacteroidetes*, is essential for host health by facilitating the metabolism of complex carbohydrate molecules and the generation of short-chain fatty acids (SCFAs) [10]. Dysbiosis—characterized by reduced microbial diversity, depletion of beneficial taxa, and functional impairment—has been more frequently connected to a number of immunological, metabolic, neurological, and cancerous conditions [11]. Interestingly, bioactive components derived from sea cucumber viscera have been demonstrated to enhance microbial diversity, elevate the relative abundance of beneficial genera such as *Lactobacillus*, *Bacteroides*, and *Akkermansia*, and increase SCFAs levels, thereby indirectly promoting host health.

Recent developments in the study of sea cucumber viscera are compiled in this review. Beginning with an overview of their principal bioactive constituents, we clarify their regulatory effects on the gut microbiota and discuss the subsequent health benefits mediated through microbiota modulation. The objective is to offer a scientific foundation for the high-value use of sea cucumber processing byproducts and to recommend new avenues for further study and applications.

## 2. Nutritional and Bioactive Components in Sea Cucumber Viscera

**Figure 1:**
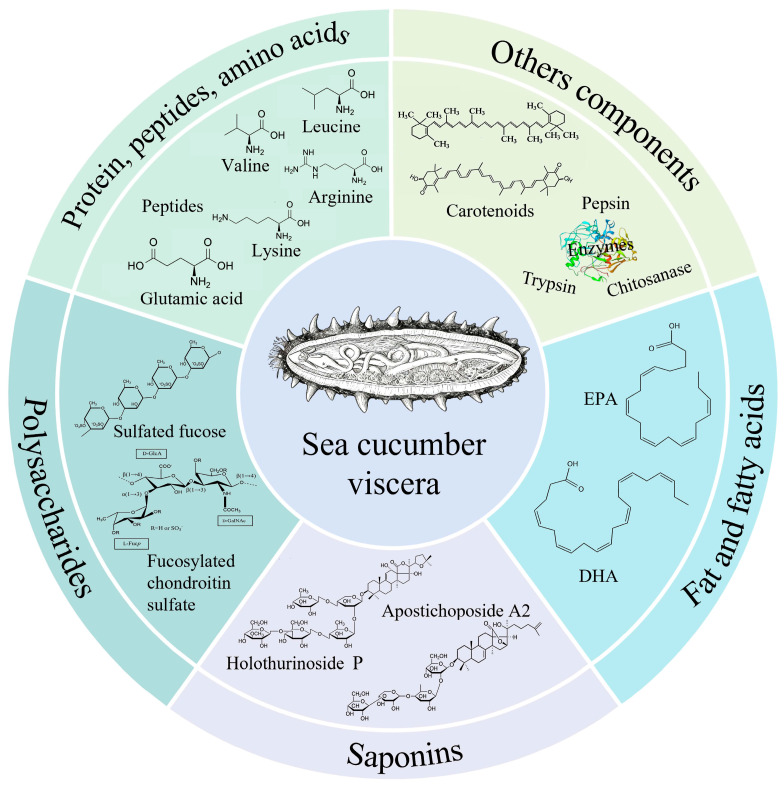
Schematic diagram of bioactive components in sea cucumber viscera.

After notable advancements in the sea cucumber manufacturing sector, the potentials of sea cucumber viscera as an important resource is being reevaluated. Researchers have explored nutrients of sea cucumber viscera and carried out the separation and purification of active substances (Figure 1). It has been found that the viscera contain various substances such as sea cucumber saponins, sea cucumber polysaccharides and various active enzymes, which have multiple biological activities such as anti-aging and lipid–lowering [12]. They have comparatively high protein compositions as well as intriguing concentrations of minerals, important trace elements, and vital amino acids [13]. Mi Rui and others compared the components of the proteolytic hydrolysates of sea cucumber body wall and viscera, they concluded that the main components of the enzymatic hydrolysates are proteins and polypeptides, accounting for 50.62% and 49.12% respectively. The total sugar contents of the two are 14.35% and 11.67% respectively, and the crude fat contents account for 10.45% and 9.64% respectively [14]. Guo Pengliu and co authours pointed out that the ingredients of the enzymatic hydrolysate powder of sea cucumber viscera are mainly 51.04% protein, 9.84% fat and 6.7% polysaccharide [15]. In addition, the edible gonads of sea cucumbers mainly contain 51.83% protein, 20.09% fat, and 18.57% carbohydrates.

### 2.1. Sea Cucumber Visceral Protein, Bioactive Peptides, Amino Acids

Protein is the main nutrient in sea cucumbers, accounting for over 70% of the total mass of the body wall. The protein content in sea cucumber viscera is also relatively high, making it a good protein source for preparing sea cucumber peptides. Yang Dongda and others analyzed the fundamental substance of sea cucumber viscera and found that the highest amount was protein, accounting for 59.50% of the total mass, while the fat content was relatively low, accounting for 7.64% of the total mass [16]. It can be seen that sea cucumber viscera is a good resource for preparing highly active sea cucumber peptides. Sea cucumbers contain an abundance of necessary amino acids, and the amino acid content varies slightly related to the area they found. Enzymatic hydrolysate powder derived from the viscera of sea cucumbers harvested in Weihai, Shandong, the proportion of the total amount of amino acids to the total mass of the enzymatic hydrolysate powder is 30.59%, the ratio of the total amount of necessary amino acids to the total amount of amino acids is 36.87%, and the ratio of necessary amino acids to non-necessary amino acids is 58.41%, which is close to the ideal model. Among them, the glutamic acid content is the highest, reaching 5.66% [15]. In the viscera-gonads of sea cucumbers from the waters of Telong Island, Batam Island, Indonesia, essential amino acids account for 49.95%, and the main amino acid types include leucine (3.81%) and arginine (4.16%) [17]. In Atlantic sea cucumbers, essential amino acids account for 37.3%, with leucine having the highest content, accounting for 7.2%, followed by lysine at 6.6% and valine at 5.4% [18].

The rich proteins in sea cucumber viscera make it an excellent raw material for extracting active peptides. Sea cucumber viscera polypeptides exhibit an extensive range of biological activities such as antioxidant, ACE inhibitory activity, anti-fatigue and anti-aging effects. The extraction of these polypeptides mostly adopts the method of exogenous enzyme hydrolysis. The types of exogenous enzymes mainly include neutral protease, alkaline protease, trypsin, flavor protease, bromelain, pepsin, papain, etc. Different enzymes and treatment conditions lead to slight differences in the extraction content of polypeptides. Yan Mingyan studied the influence of enzyme types on sea cucumber viscera hydrolysates. The results showed that alkaline protease, trypsin and flavor protease were successful in producing peptides having substantial antioxidant capacity, and they were also effective in generating smaller peptide fragments [19]. According to Qin Hong, the key parameters affecting the degree of enzymatic hydrolysis were identified, in descending order of influence, as: substrate concentration > enzyme dosage > temperature > time [20]. Table 1 summarizes different treatment conditions for sea cucumber viscera polypeptides.

### 2.2. Sea Cucumber Visceral Polysaccharides

Similarly to the body wall, the viscera of sea cucumbers include polysaccharides as key bioactive components, predominantly distributed in the intestinal wall, respiratory tree, oocytes, and sperm cells [3]. The acidic polysaccharides obtained from sea cucumbers primarily comprise two structural classes: sulfated fucans and fucosylated chondroitin sulfate (FCS), the latter characterized by distinct sulfation patterns on its fucose side chains. Recent studies involving the separation and extraction of polysaccharides from the intestinal tract of *Apostichopus japonicus* indicate, based on structural analyses, that these compounds belong to the chondroitin sulfate family [27]. Previous studies have shown that the polysaccharides separated and extracted from the viscera of sea cucumbers have anti-tumor activity, antioxidant activity, and good inhibitory ability against human prostate cancer [28,29,30]. Yang Dongda purified a sulfated polysaccharide from sea cucumber viscera with a distinct monosaccharide composition: the molar ratios of mannose, glucosamine, glucuronic acid, N-acetyl-galactosamine, glucose, galactose, and fructose are 1.00:1.41:0.88:2.14:1.90:1.12:1.24 [27]. Similarly, Zhang Zhuchi pointed out that the polysaccharides of sea cucumbers viscera have potential immunomodulatory activity in cyclophosphamide-induced mice [31].

### 2.3. Sea Cucumber Visceral Saponins

When sea cucumbers are stimulated by the external environment, the nervous system releases a kind of neurotoxin, which is called sea cucumber saponin, also known as holothurin. It is an important and abundant secondary metabolite of sea cucumbers. Research shows that saponin content varies significantly in several sea cucumber sections. The saponin content in the body wall is about 0.37%, about 1.03% in the intestinal wall of the viscera, and about 1.65% in the respiratory tree. Sea cucumber-derived saponins are predominantly triterpenoid glycosides that fall into the category of holostane-type structures. However, Zhang et al. found two novel saponins isolated from sea cucumber viscera. Both of these isolated saponins are characterized by non-holostane steroid glycosides [32]. Currently, more than 250 triterpenoid glycosides from different sea cucumber species have been reported. They are predominantly classified into three holostane-type groups according to their aglycone moieties: those with 3β-hydroxyholost-9(11)-ene, 3β-hydroxyholost-7-ene, or other holostane skeletons, alongside a fourth category comprising non-holostane aglycones [33]. The research by Yadollah and his colleagues pointed out that the viscera are the main source of saponins. At least 39 new saponins with high variability in structure and a further 36 reported triterpenoid glycosides with various aglycone and glycosyl moieties were successfully characterized by their work from the viscera of the Australian sea cucumber Holothuria lessoni Massin. Compared with the body wall, the compounds in the visceral samples have higher diversity and yield [33]. Analysis by Philippe et al. via mass spectrometry revealed the presence of 26 sulfated saponins (with five elemental compositions) in *H. scabra* viscera [34]. Their cytotoxicity was directly attributed to the sulfate groups. These findings align with broader research confirming that sea cucumber saponins possess diverse bioactivities, including hemolytic, immunomodulatory, anti-tumor, anti-cancer, and anti-diabetic effects [35].

### 2.4. Sea Cucumber Visceral Fat and Fatty Acids

Sea cucumber viscera are an important source of various fatty acids, being particularly abundant in polyunsaturated fatty acids (PUFAs) with significant biological activity. The freeze-dried powder of *Cucumaria frondosa* has been characterized by the presence of nine major lipid classes, which include hydrocarbons, ethyl ketones, alcohols, triacylglycerols (TAGs), monoesters, free fatty acids (FFAs), sterols, and phospholipids (PLs) [36]. Notably, fatty acids are not only abundant in total quantity but also exhibit specific molecular forms, with phospholipids serving as one of their primary carriers. Multiple analyses confirm this profile; for instance, unsaturated fatty acids in the viscera of *Apostichopus japonicus* can comprise up to 75.64% of total fatty acids, with health-relevant PUFAs accounting for 45.44% [15]. Eicosapentaenoic acid (EPA) is particularly prominent, representing up to 24.4% of total fatty acids in certain studies—significantly higher than levels found in corresponding body-wall samples [37,38]. Similarly, work by Lin et al. on lipid extraction from *C. frondosa* viscera reported total fatty acid contents of 9.02–17.43 g/100 g (dry weight), of which omega-3 fatty acids constituted 2.22–3.92 g/100 g [5]. However, such enrichment patterns are species-specific. For example, in *Holothuria atra*, both EPA and docosahexaenoic acid (DHA) levels are higher in the body wall than in the viscera: EPA reaches 8.56% and DHA 1.6% in the body wall, compared to only 0.1% EPA and 0.37% DHA in the viscera [39]. Interestingly, seasonal variation also influences the visceral lipid profile. Abuzaytoun et al. observed higher lipid content in winter, with diacylglycerol ethers (DAGE) comprising 55% of total lipid extract mass, and 12-MTA accounting for 42% of total fatty acids within DAGE. In summer, TAG content rose to 44%, exceeding that of 12-MTA, while EPA became more abundant (constituting 20% of total fatty acids) [40]. Moreover, 12-MTA represented only 4–9% of total fatty acids in fresh or rehydrated whole-body (body wall plus viscera) powder of *C. frondosa* [41]. These highly unsaturated fatty acids—especially EPA in phospholipid form—serve not only as essential precursors for cell membranes and signaling molecules, but may also confer potential benefits for host nervous system health. Such effects likely arise from restoration of the balance of the gut microbiota, reinforcement of the intestinal mucosal barrier, regulation of local immunity, and subsequent gut–brain axis communication.

### 2.5. Other Bioactive Components: Pigments, Flavonoids, and Enzymes

In addition to nutrients such as polysaccharides, saponins, polyphenols, and fatty acids, the viscera of sea cucumbers also contain components such as pigments, flavonoids, and enzymes.

In the viscera of sea cucumbers, especially in the gonads, there are various carotenoids, mainly astaxanthin and canthaxanthin. The composition changes according to the species, geographical location, and body parts. Investigations have confirmed the presence of several carotenoids—such as astaxanthin, zeaxanthin, lutein, and β-carotene—within sea cucumber gonads. Studies have shown that the amount of carotenoid in the crude extract of sea cucumber gonads reaches 71.62 μg/g, and the total carotenoid content in sea cucumber eggs reaches 113 μg/g [42]. From the gonads of *Cucumaria japonica*, Tsushima et al. isolated and characterized three carotenoids with the 5,6,5′,6′-tetrahydrocarotene structure (9Z,9′Z configuration), designating them cucumariaxanthins A, B, and C. Notably, cucumariaxanthin C exhibited specific antiviral activity against the activated Epstein–Barr virus [43].

The viscera of sea cucumbers have good antioxidant activity, and this activity is closely related to the phenolic and flavonoid substances in the viscera. In addition, the viscera of sea cucumbers contain a variety of digestive and metabolic enzymes, including chitosanase, pepsin, trypsin, alkaline phosphatase, etc. These enzymes not only play a role in their own nutrient digestion but may also, during processing, generate peptides or oligosaccharides with specific bioactivities through enzymatic hydrolysis, thereby indirectly contributing to their overall bioactive profile.

Collectively, sea cucumbers possess significant nutritional potential, serving as a valuable source of diverse bioactive compounds encompassing amino acids, triterpene glycosides, polyunsaturated fatty acids, polysaccharides, carotenoids, phenols, flavonoids, and bioactive peptides.

## 3. Modulation of Gut Microbiota by Sea Cucumber Viscera Components

**Figure 2:**
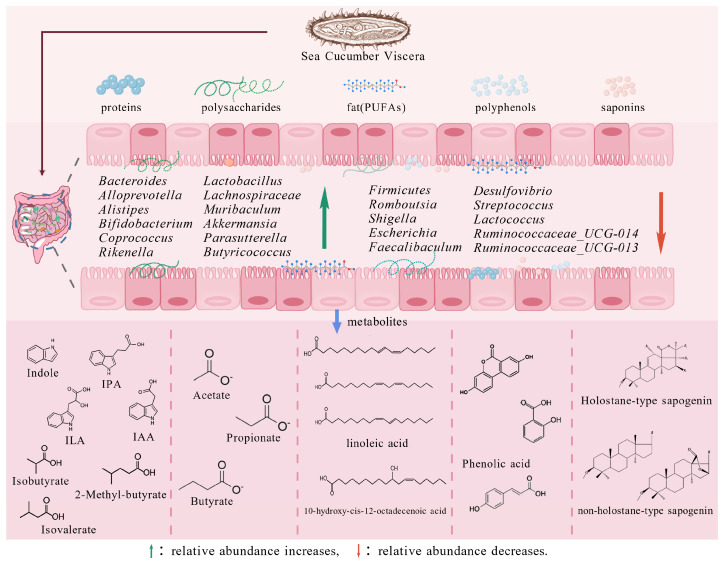
Regulation of gut microbiota and beneficial metabolites by sea cucumber viscera.

The gut microbiota is a complex microbial ecosystem that colonizes the animal digestive tract. These symbiotic microorganisms exert critical regulatory influences on host physiology—including digestion, immunity, and disease resistance—through their roles in nutrient metabolism, immune modulation, and barrier maintenance. A state of dysbiosis is linked to various chronic conditions, such as metabolic syndrome, obesity, diabetes, and intestinal inflammation. Recent studies indicate that sea cucumber viscera, particularly bioactive components like enzymatic hydrolysates and polysaccharides, can modulate the gut microbiota and influence metabolite production (Figure 2).

### 3.1. Changes in Gut Microbiota Composition

The gut microbiota plays an important role in preserving the gut mucosal barrier to prevent pathogenic agents from entering systemic circulation through mucosal tissues. It has been proven that sea cucumber viscera can regulate the overall structure of the gut microbiota and restore microbial homeostasis, which is summarized in Table 2. The relative abundances of Bacteroidetes, Firmicutes, and Proteobacteria in the gut microbiota exceed 90%, and the intake of sea cucumber viscera components can regulate the relative abundances of these phyla. Researchers have found that the visceral enzymatic hydrolysate leads to the reconstruction of the relative abundance of *Bacteroidetes* and *Firmicutes*, which are the core gut microbial communities, and their dynamic balance is intimately associated with the host’s metabolic wellness. Meanwhile, the intake of viscera enzymatic hydrolysate could boost the relative number of advantageous genera like as *Dubosiella*, *Lactobacillus*, *Bacteroides*, and *Akkermansia*, which are related to glycogen storage, immune regulation, and anti-inflammation. Bai Yating pointed out that the supplementation of sea cucumber gonad enzymatic hydrolysate significantly enhances the variety of gut microbiota in mice and tilapia, including several distinct bacterial populations in the tilapia gut [44]. Among them, the relative abundance of *Lactobacillus*, *Ruminococcus*, *Akkermansia*, *Roseburia*, and an unclassified genus of *Erysipelotrichaceae* in the mouse cecum increased. *Lactobacillus*, capable of producing organic acids, bacteriocins, hydrogen peroxide, and diacetyl, contributes antibacterial functions [45]. *Ruminococcus* can produce a large amount of cellulase and hemicellulase [46]. *Akkermansia* has been proven to significantly improve the body’s glucose metabolism [47]. *Roseburia* contributes to host physiology through its production of SCFAs. These metabolites exert pivotal effects on the metabolism, growth, differentiation, and blood supply of cells in both the colon and small intestine [48]. In a murine model of ulcerative colitis, supplementation with sea cucumber body wall peptides ameliorated gut microbiota dysbiosis. This was manifested by an increased abundance of beneficial taxa such as the *Lachnospiraceae* NK4A136 group, *Prevotellaceae* UCG-001, and *Ligilactobacillus*, alongside a reduction in *Bacteroides* and the *Eubacterium ruminantium*. Concurrently, the treatment alleviated the colitis-associated reduction in fecal short-chain fatty acid level [49]. Gong et al.’s research once again confirmed that sea cucumber egg hydrolysate could also reduce intestinal inflammation, which may be because the sea cucumber egg hydrolysate contains the same polypeptide components [50]. Polysaccharides in sea cucumber viscera, especially sulfated polysaccharides, as dietary fibers that are not easily digested by the host, can safely reach the distal intestine and become fermentation substrates for gut microbiota, selectively enhancing the growth of certain beneficial microbial communities. Sulfated polysaccharides derived from sea cucumbers have been shown to enhance the development of specific gut bacteria, such as Bacteroides and Prevotella, known for their high efficiency in degrading complex polysaccharides [51]. These bacteria encode abundant carbohydrate—active enzymes (CAZymes) that can specifically hydrolyze the complex glycosidic bonds in sea cucumber polysaccharides and utilize them for energy production [52]. Moreover, sea cucumber-derived Fuc promotes the growth of SCFA-producing bacteria, including *Coprococcus*, *Rikenella*, and *Butyricococcus*, facilitating intestinal mucosal repair and overall gut health [53]. Cao et al., through in vivo experiments, provided evidence that fucoidan isolated from sea cucumber cooking liquid restores the relative abundance of *Akkermansia* and *Lactobacillus*. They suggested that this polysaccharide may alleviate *Helicobacter pylori*-induced gastritis, in part, by modulating such key microbial communities [54].

### 3.2. Microbial Metabolites Derived from Sea Cucumber Viscera

Variations in gut microbial composition can induce metabolic shifts, thereby influencing host phenotype [57]. The viscera of sea cucumbers include proteins, polysaccharides, saponins, fatty acids, enzymes, and polyphenols, which are metabolized by the intestinal flora into various products that confer health benefits to the host (Figure 2).

Polysaccharides represent the most extensively studied class of compounds in sea cucumber research. The gut microbiota degrades complex carbohydrates to generate SCFAs, which serve as a supply of energy for the individual. It is essential in sustaining host health, immunity, nutrient metabolism, neuroregulation, and intestinal barrier integrity [58]. Polysaccharides from sea cucumber viscera are resistant to host digestion and transit intact to the colon, the local bacteria use them as digestion substrates and ultimately metabolize them into SCFAs, primarily acetate, propionate, and butyrate [59]. Among microbial metabolites, acetate serves as a key energetic mediator in colon-host crosstalk, primarily produced by dominant anaerobic bacteria such as *Lactobacillus* through fermentation of carbohydrates not fully absorbed in the small intestine [60]. It serves not only as a precursor for cholesterol biosynthesis but also supplies approximately 10% of the host’s daily energy, representing a central vector for metabolic energy transfer from the gut microbiota to the host [61]. Propionate, a colon-derived metabolite, is closely linked to hepatic cholesterol biosynthesis. Following transepithelial transport in the colon, it is delivered via the portal vein to the liver. Within hepatocyte mitochondria, propionyl-CoA carboxylase mediates its entry into the pyruvate–oxaloacetate–phosphoenolpyruvate metabolic axis, thereby driving gluconeogenesis [62,63]. Butyrate, primarily produced by bacteria of the phylum Firmicutes, acts as the major energy substrate for colonocytes. Although much of the existing evidence derives from studies on sea cucumber body wall components, it is noteworthy that the visceral components share similarities, differing mainly in the content and profile of bioactive constituents across tissues.

Proteins constitute a considerable proportion of sea cucumber viscera. Approximately 90% of dietary proteins are absorbed in the small intestine, while the undigested portion reaches the colon to serve as a substrate for microbial metabolism. Protein degradation in the gut is mainly facilitated by bacteria such as *Bacteroides*, *Fusobacterium*, and *Streptococcus*. Through microbial deamination and decarboxylation, proteins yield metabolites including indole derivatives, ammonia, phenols, and amines [64]. Specifically, gut microbiota such as *Clostridium*, *Bacteroides*, and *Bifidobacterium* are capable of transforming tryptophan into a series of indole derivatives, including IAA, IPA, indole-3-ethanol (IEt), indole-3-acrylic acid (IArA), and indole-3-aldehyde (IAld) [65]. These resultant metabolites serve as endogenous ligands for the aryl hydrocarbon receptor (AhR) in the intestine, thereby regulating critical processes for homeostasis, such as epithelial renewal, barrier integrity, and immune cell population dynamics [66,67]. Saponins are progressively deglycosylated by gut microbiota including *Bifidobacterium*, *Lactobacillus*, *Bacteroides*, and *Prevotella*, yielding a series of secondary saponins and ultimately aglycones [68]. The aglycones may further undergo hydroxylation, dehydrogenation, dehydration, and demethylation mediated by intestinal bacteria. Lipids in sea cucumber viscera are predominantly PUFAs. Following ingestion, these fatty acid precursors are endogenously metabolized through host synthesis pathways, yielding either dihomo-γ-linolenic acid (DGLA) and arachidonic acid (AA), or EPA and DHA. Gut microbes also biotransform linoleic acid (LA) and α-linolenic acid (LNA) derived from lipid hydrolysis, altering the position of carbon-carbon unsaturated bonds to produce a range of microbially derived fatty acid isomers [69]. For instance, the hydroxy derivative of LA, 10-hydroxy-cis-12-octadecenoic acid (HYA), has been shown to activate the free fatty acid receptors GPR40 and GPR120 in the gut [70]. HYA-GPR40/GPR120 signaling subsequently promotes the secretion of intestinal glucagon-like peptide-1 (GLP-1), thereby modulating host metabolic homeostasis. Additionally, sea cucumber viscera contain small amounts of polyphenols. Studies indicate that about 90% of phenolic compounds require gut microbial involvement for metabolism, ultimately being converted into low-molecular-weight phenolic acids [71]. Phenolic acids have been demonstrated to modulate intestinal immune responses and contribute to gut health while mitigating metabolic dysfunction.

Recent evidence indicates that colon tissues of mice fed sea cucumber egg hydrolysates exhibit elevated levels of metabolites such as phenyllactic acid, phenyl acetate, uracil, and 7-methylguanine, which are associated with enhanced production of anti-inflammatory compounds and suppression of systemic inflammation [50]. Zhu Zhenjun et al. reported that mice administered sea cucumber polysaccharides showed increased levels of acetate, propionate, butyrate, and total SCFAs compared to a normal diet group, with acetate being the principal product of polysaccharide fermentation [72]. SCFAs are essential for immunological control and maintenance of intestinal barrier integrity. They stimulate intestinal epithelial cells to release IL-18, antimicrobial peptides, and mucus, thereby reinforcing barrier function. Intervention with sea cucumber fucoidan increased the abundance of *Prevotellaceae* UCG-001, *Alloprevotella*, the *Eubacterium coprostanoligenes* group, and *Oscillospiraceae* [54]. Notably, the *Lachnospiraceae* NK4A136 group—widely recognized as a SCFA producer—was associated with elevated levels of isobutyrate, butyrate, isocaproate, and caproate [73]. Furthermore, sea cucumber polypeptides demonstrate comparable healthy effect. Interestingly, their effect on SCFA production appears to follow a non-linear dose–response relationship, with low-dose supplementation (200 mg/kg·bw) promoting SCFA yield more effectively than medium- or high-dose regimens (500 and 1000 mg/kg·bw) [49]. Nevertheless, in existing research on sea cucumber viscera, few studies have isolated individual components to systematically investigate their metabolic pathways.

## 4. Gut Microbiota-Mediated Health Benefits of Sea Cucumber Viscera

**Figure 3:**
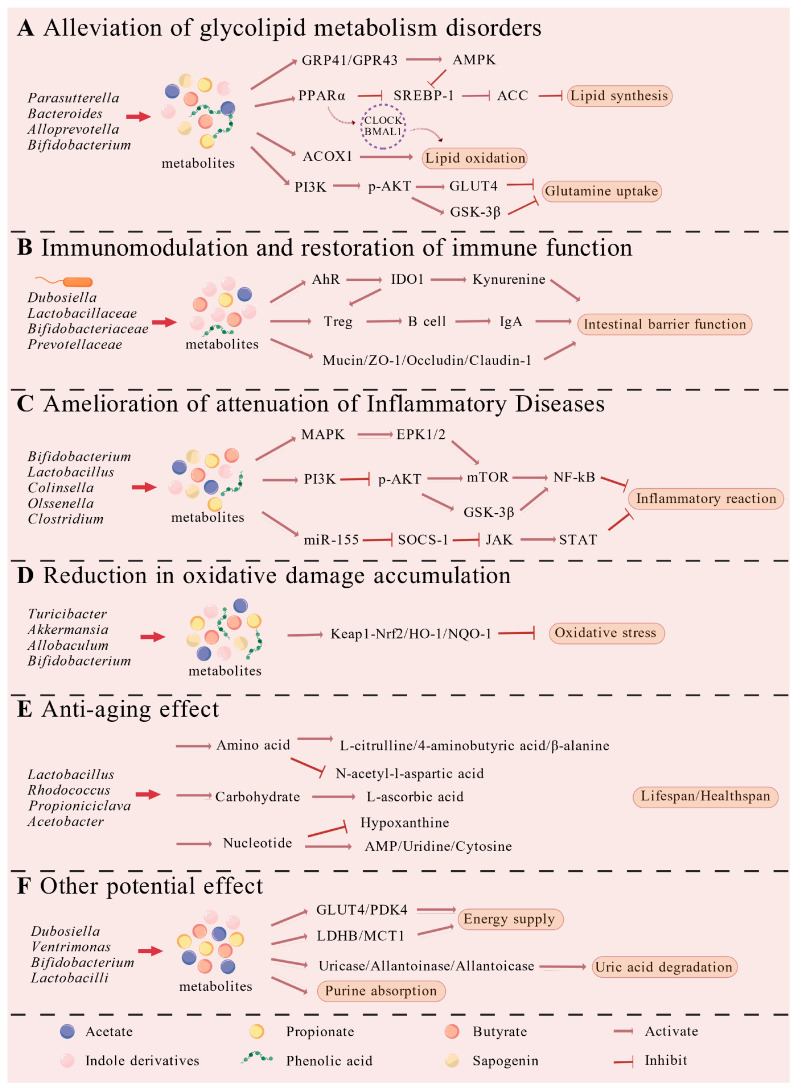
Gut microbiota-mediated mechanisms of sea cucumber viscera in disease intervention. (**A**) Alleviation of glycolipid metabolism disorders; (**B**) Immunomodulation and restoration of immune function; (**C**) Amelioration of inflammatory diseases; (**D**) Reduction in oxidative damage accumulation; (**E**) Anti-aging effect; (**F**) Other potential benefits.

Recent research indicates that bioactive components derived from sea cucumber viscera are metabolized by the gut microbiota into products such as SCFAs, indole derivatives, and phenolic acids. These metabolites, in turn, mediate potential physiological benefits, including improved metabolic health, modulated immune and inflammatory responses, as well as antioxidant, anti-fatigue, and anti-aging syndrome activities [74]. This section will detail how the gut microbiota and metabolic profiles modulated by visceral bioactives contribute to these specific host health improvements (Figure 3).

### 4.1. Alleviation of Glycolipid Metabolism Disorders

Obesity, a prevalent metabolic disorder, is frequently associated with numerous health complications. The critical involvement of gut microbiota in its pathogenesis and in the emergence of associated metabolic dysfunctions has been progressively demonstrated by recent data. In close connection, type 2 diabetes mellitus (T2DM) accounts for around 90% of all instances of diabetes mellitus, which is a significant chronic metabolic illness [75]. The primary pathological features of T2DM include insulin resistance, dysregulated glucose metabolism, and impaired lipid metabolism [76]. Studies indicate that components derived from sea cucumber viscera can act in concert through dual pathways—a gut microbiota-dependent mechanism and direct molecular interactions with host tissues—to collectively modulate glucose homeostasis and lipid metabolism, thereby ameliorating glucose and lipid metabolic disorders.

#### 4.1.1. Gut Microbiota-Dependent Mechanisms

It is known that altering the gut microbiota and its beneficial metabolites is a useful tactic for preventing obesity [77]. Considering the chemical similarity between sea cucumber gonads and the body wall, the demonstrated role of body wall components in regulating glucose and lipid metabolism supports the potential for visceral constituents to exert analogous effects. Evidence from multiple studies indicates that bioactive extracts from the sea cucumber body wall demonstrate beneficial effects on blood glucose regulation in diabetic animal models [78]. Among them, the genus *Akkermansia* has been shown to significantly improve systemic glucose metabolism and to possess preventive and therapeutic potential against conditions such as hyperglycemia [79]. Bai et al. reported that supplementation with enzymatic hydrolysates of sea cucumber viscera promotes an increase in the relative abundance of *Akkermansia* [44].

Notably, long-chain bases derived from sea cucumbers have been shown to counteract the high-fat-diet-induced increase in the *Firmicutes*/*Bacteroidetes* ratio [80]. They achieve this by decreasing the abundance of several *Firmicutes* (including *Ruminococcaceae_*UCG-014, *[Ruminococcus]_torques_group*) and increasing beneficial Bacteroidetes (such as *Alloprevotella*, *Rikenellaceae_RC9_gut_group*), an effect associated with elevated SCFAs [75]. The resulting SCFAs help balance opposing metabolic processes like lipogenesis and lipolysis through receptors GPR41 and GPR43 [81]. Substantial evidence associates the upregulation of GPR41 and GPR43 with the inhibition of obesity and its related metabolic complications [82]. Additionally, prebiotic supplementation in high-fat diet-fed mice can reverse the Firmicutes/Bacteroidetes ratio, thereby preventing diet-induced obesity. Sulfated polysaccharides from sea cucumbers reduce body weight, adipose and hepatic hypertrophy, insulin resistance, and serum levels of lipids and inflammatory cytokines in high-fat diet-fed mice. Specific microbial shifts, including strong positive correlations between *Faecalibaculum*, *Desulfovibrio*, *Streptococcus*, and *Lactococcus* with obesity symptoms, and between *Alistipes*, *Ruminococcaceae_*UCG-014, *Bifidobacterium*, and *Candidatus_Saccharimonas* with intestinal tissue indices, suggest that sea cucumber polysaccharides prevent diet-induced obesity and related disorders by modulating gut microbiota, improving microbial metabolites, and enhancing intestinal integrity. Notably, the anti-obesity efficacy of these polysaccharides can be further enhanced through free-radical depolymerization [83].

#### 4.1.2. Direct Action Pathways

Direct evidence indicates that hydrolysates of sea cucumber gonads activate the PI3K/Akt and AMPK signaling mechanisms. This activation increases the production of GSK-3β and ACC proteins, which promote lipid metabolism and insulin susceptibility [75]. Moreover, Nguyen et al. purified 1,3-dipalmitoyl and cis-9-octadecenoic acid from sea cucumber viscera, both exhibiting potent α-glucosidase inhibitory activity. Mammalian α-glucosidase is the primary enzyme responsible for the last phase of carbohydrate digestion, releasing absorbable sugars. By inhibiting this enzyme, the liberation of oligosaccharides, disaccharides, and D-glucose from complex carbohydrates is delayed. Consequently, intestinal glucose absorption is slowed, thus helping to suppress postprandial hyperglycemia in individuals with diabetes [84]. This suggests that sea cucumber viscera may serve as a novel source of α-glucosidase inhibitors, suitable for preventing obesity and diabetes.

Indirect evidence suggests that bioactive components in sea cucumber viscera could be processed by gut bacteria to produce favorable metabolites, displaying impact on regulation on glucose and lipid metabolism. Sulfated polysaccharides extracted from sea cucumbers have been shown to have anti-diabetic effects. One study looked at the anti-hyperglycemic properties of fucoidan extracted from *Cucumaria frondosa*. The results demonstrated that fucoidan stimulates the PI3K/PKB pathway and upregulates *GLUT4* mRNA expression, while increasing the phosphorylation of PKB and PI3K [85]. These actions contribute to significant anti-hyperglycemic effects in skeletal fat and muscle. The PI3K/PKB mechanism is a major insulin signaling cascade that governs glucose metabolism [86,87], and GLUT4 is the primary glucose transporter controlled by this route [88]. Deficiencies in these compounds can result in insulin resistance.

Furthermore, compared with other sea cucumber components, saponins appear to exhibit superior lipid-lowering activity. Sea cucumber saponins exert anti-lipidemic effects in SD rats and C57BL/6 mice through a mechanism involving the inhibition of SREBP-1c to reduce lipogenesis and the enhancement of PPARα and acyl-CoA oxidase 1 (ACOX1) expression to promote fatty acid β-oxidation, collectively ameliorating lipid deposition [83,89]. Simultaneously, supplementation with sea cucumber saponins may regulate lipid metabolism by modulating circadian clock genes such as *CLOCK* and *BMAL1* in ICR mice, as reflected in rhythmic expression changes in lipid metabolism-related genes including *PPARα*, *SREBP-1c*, *CPT*, and *FAS* [90].

It is important to emphasize that gut microbiota-dependent mechanisms and direct molecular action pathways collectively form a synergistic and potentially amplifying network. On one hand, bioactive components from the viscera can directly regulate key signaling pathways within host cells, including AMPK and PPAR. On the other hand, these components are metabolized by the gut microbiota into beneficial metabolites such as SCFAs, which subsequently act as ligands to indirectly activate the same signaling pathways. Through these coordinated actions, they collectively regulate glucose and lipid metabolism. In summary, active constituents from sea cucumber viscera can reshape the gut microbial composition and alleviate host metabolic disorders—such as diabetes, obesity, and hyperlipidemia—caused by dysregulated glucose and lipid metabolism through multiple integrated mechanisms.

### 4.2. Immunomodulation and Restoration of Immune Function

The immune system of the body is a host protection mechanism made up of many immune cells, tissues, and organs that work together to destroy potentially dangerous chemicals and aberrant cells. Given its role as a complex and multifunctional ecosystem crucial for maintaining host health—encompassing nutrient metabolism, pathogen defense, and modulation of immune responses—the gut microbiota has attracted growing interest as a target for interventions aimed at enhancing immune resilience and optimizing nutrient metabolism. Notably, research indicates that sea cucumber-derived sulfated polysaccharides demonstrate potential for COVID-19 prevention, as evidenced by their potent in vitro inhibition of SARS-CoV-2 [91].

Studies in Balb/c mice have demonstrated that adding sea cucumber visceral polysaccharides to the diet increases the relative abundance of advantageous genera (*Lactobacillus*, *Bacteroides*, and *Akkermansia*). The families to which these genera belong, *Lactobacillaceae*, *Bifidobacteriaceae*, and *Prevotellaceae*, are recognized for their role in resisting pathogenic colonization and boosting intestinal barrier function through the promotion of secretory immune responses. Furthermore, polysaccharide components from sea cucumber viscera have been found to induce the proliferation of T cells and B cells in murine splenic lymphocytes and to enhance the proliferative capacity and phagocytic activity of macrophages [31].

Emerging research highlights *Dubosiella* for its involvement in tryptophan and lysine metabolism, thereby linking amino acid processing to immune regulation through the L-lysine–AhR–IDO1–kynurenine pathway. Direct evidence indicates that hydrolysates derived from sea cucumber intestines and eggs significantly increase the relative abundance of *Dubosiella* and elevate levels of SCFAs [55]. Among SCFAs, butyrate is known to promote immunosuppressive regulatory T cell development, a key immunosuppressive population that curbs excessive inflammatory responses and maintains intestinal immune tolerance. Complementarily, a broader array of microbial metabolites function as signaling molecules, stimulating the secretion of antimicrobial effectors like peptides and secretory IgA from epithelial and innate immune cells, thereby directly enhancing mucosal defense.

In summary, polysaccharides derived from the internal organs of sea cucumbers are likely to mediate immunomodulatory effects via interactions with the gut microbiota. The potential immunological roles of other bioactive constituents—such as proteins, saponins, and fatty acids—warrant further investigation and validation through additional in vivo studies.

### 4.3. Amelioration of Inflammatory Diseases

Inflammation represents a typical host response to infection; however, excessive or dysregulated inflammation can exacerbate autoimmune and chronic inflammatory diseases. According to earlier research, the synthesis of SCFAs and the makeup of the gut microbiota are directly related to the release of inflammatory cytokines. Because sea cucumbers are rich in both necessary and non-essential amino acids, a recent study indicates that bioactive chemicals isolated from them, such as polysaccharides and peptides, may have anti-inflammatory qualities [92]. For instance, a peptide fraction (molecular weight 180–1000 Da, purity 72.12%) obtained from sea cucumber via enzymatic hydrolysis effectively suppressed lipopolysaccharide-induced inflammation and upregulated *HO-1* mRNA expression in RAW264.7 macrophages [93]. Hu et al. demonstrated that dietary intake of chondroitin sulfate extracted from sea cucumbers markedly altered the gut microbiota profile, characterized by a reduction in *Bacilli*, an increase in *Firmicutes*, enrichment of beneficial taxa such as *Lactobacillus* (a gut barrier protector) and SCFA-producing bacteria (e.g., *Lactobacillus*, *Bifidobacterium*, and the *Lachnospiraceae* NK4A136 group), and a decrease in lipopolysaccharide (LPS)-producing bacteria (e.g., *Escherichia coli*). These microbial shifts inhibited inflammatory responses, lowered serum and fecal LPS levels, suppressed the transcription of Toll-like receptor 4 and its downstream proteins, elevated fecal SCFA concentrations, and activated AMPK, collectively contributing to anti-inflammatory effects [94].

Direct evidence for the anti-inflammatory activity of sea cucumber viscera comes from a study showing that hydrolysate of sea cucumber eggs increased the relative abundance of *Muribaculum* while suppressing *Shigella* and *Escherichia* in C57 mice. These microbiota changes induced shifts in the metabolomic profile, influencing key metabolic processes, such as amino acid production, central conversion of carbon, ABC transporter function, protein digestion and absorption, mineral absorption, and the metabolism of D-amino acids and linoleic acid [50]. Notably, levels of metabolites such as phenyllactic acid, phenyl acetate, uracil, and 7-methylguanine were elevated, indicating that sea cucumber viscera can promote the production of anti-inflammatory compounds by modulating gut microbial diversity [50]. Polysaccharides from sea cucumber viscera exert dual modulatory effects. On one hand, they upregulate the expression of immune cytokines, including IL-6, TNF-α, and IFN-γ. On the other hand, they enhance intestinal barrier integrity by increasing the protein levels of key tight junction components (claudin-1, occludin, ZO-1) and the mucin MUC2. Moreover, hydrolysates derived from both intestinal and egg tissues of sea cucumbers reduced the relative abundance of the pro-inflammatory genus *Romboutsia* [55].

According to research, the anti-inflammatory properties of sea cucumber polysaccharides are mostly dependent on how they react with gut flora. Sea cucumber-derived polysaccharides such as fucoidan and chondroitin sulfate—primarily found in the body wall and viscera—contain approximately 30% sulfate esters, distinguishing them from other polysaccharides and enhancing their bioactivity. Fucoidan from sea cucumbers promotes the proliferation of SCFA-producing bacteria, including *Coprococcus*, *Rikenella*, and *Butyricococcus*, thereby supporting intestinal mucosal repair and overall gut health. Body wall fucoidan has been shown to reverse antibiotic-induced depletion of *Lactobacillus*, significantly increase SCFA levels, modulate the MAPK/NF-κB signaling pathway, downregulate pro-inflammatory cytokines such as IL-1β and TNF-α, and regulate the expression of S100A8 and E-cadherin, ultimately alleviating *Helicobacter pylori*-induced gastritis [54]. Similarly, a branched fucoidan sulfate from *Stichopus japonicus* exhibited anti-inflammatory activity in the livers of LPS-challenged mice by downregulating targets within the MAPK/NF-κB and AKT/mTOR pathways, as well as reducing iNOS expression [95].

Previous studies have reported that sea cucumber proteins and peptides can ameliorate ulcerative colitis through anti-inflammatory mechanisms and gut microbiota modulation. Sea cucumber (*Stichopus japonicus*) major yolk protein increased the relative abundance of beneficial genera such as *Bifidobacterium*, *Lactobacillus*, *Collinsella*, *Eubacterium*, *Enterorhabdus*, *Olsenella*, *Acetatifactor*, and *Clostridium* in C57 mice, elevated colonic SCFA content, and enhanced metabolic functions related to carbohydrates, energy, cofactors, vitamins, amino acids, terpenoids, and polyketides. Consequently, secretion of anti-inflammatory cytokines (IL-4 and IL-10) was significantly increased in colitis mice. Notably, except for TNF-α, various protein doses did not affect other pro-inflammatory cytokines (TGF-β, IFN-γ, and IL-6), suggesting that their primary mode of action involves augmenting anti-inflammatory factor release [96]. Similarly, Mao et al. demonstrated that sea cucumber peptides alleviated colitis through reversing gut microbiota dysbiosis—increasing *Prevotellaceae* and *Alloprevotella* abundance and SCFA production—restoring gut barrier integrity via upregulation of Claudin-1 and Occludin, and reducing inflammation through inhibition of IL-1β, IL-6, and TNF-α and modulation of the miR-155/SOCS1 axis [49].

Collectively, this evidence indicates that various bioactive components from sea cucumber viscera can mitigate gut microbiota dysbiosis associated with inflammatory conditions. Their anti-inflammatory benefits are achieved via enrichment of beneficial bacteria, increased SCFA production, and modulation of signaling pathways including MAPK/NF-κB and the miR-155/SOCS1 axis.

### 4.4. Reduction in Oxidative Damage Accumulation

Oxidative stress constitutes a pathological state that occurs when the generation of reactive oxidants (ROS and RNS) exceeds the scavenging ability of the endogenous antioxidant system. The consequent oxidative damage to cellular components is a key contributor to the development and progression of various disorders [97,98]. Accumulating evidence indicates that diverse bioactive compounds present in sea cucumbers—including polysaccharides, proteins, and saponins—confer significant antioxidant properties. As the compositional profile of the viscera resembles that of the body wall, the intestines and gonads of sea cucumbers may have antioxidant effects through the gut–liver axis. Specifically, following microbial metabolism, bioactive constituents from sea cucumber viscera yield metabolites such as SCFAs, indole derivatives, phenolic acids, and secondary aglycones. These compounds are transported via the portal circulation to the liver, where they orchestrate systemic modulation of the hepatic antioxidant defense network through activation of key signaling axes including Nrf2 and AhR. Dietary supplementation with sea cucumber viscera increases the relative abundance of intestinal bacteria such as *Turicibacter*, *Akkermansia*, *Allobaculum*, and *Bifidobacterium*, which are known to modulate intestinal barrier thickness and maintain its integrity [99,100]. Evidence indicates that hydrolysates of sea cucumber viscera can selectively enrich beneficial intestinal bacteria, including *Muribaculum*, in murine models. This modulation regulates host amino acid metabolism and elevates circulating levels of microbiota-derived metabolites, such as phenyllactic acid and phenyl acetate [50]. Following absorption into the systemic circulation, these metabolites directly influence host cellular metabolism. Corroborating this, studies employing Caco-2 cell models demonstrate that fermented viscera hydrolysates significantly enrich key amino acid metabolic pathways—including those for arginine, alanine, aspartate, and glutamate—and comprehensively upregulate both glucose and amino acid metabolism [101]. This remodeling of the intracellular metabolic network provides the material and energy foundation necessary for activating the core antioxidant defense system. Consequently, it specifically triggers the Nrf2/HO-1 signaling pathway, upregulates the expression of antioxidant enzyme genes such as *HO-1* and *NQO-1*, and ultimately leads to a systemic enhancement of antioxidant capacity while mitigating oxidative damage and cell apoptosis [101]. Mechanistic insights from these in vitro models gain direct support from in vivo studies. Research shows that oral administration of lyophilized sea cucumber viscera powder exerts significant antioxidant effects in mice, evidenced by reduced serum malondialdehyde levels and increased hepatic antioxidant enzyme activity. This protective effect is mediated primarily via activation of the Keap1–Nrf2/HO-1 pathway, with a dose of 400 mg/kg/day demonstrating superior efficacy compared to 200 and 800 mg/kg/day regimens [56].

Together with previously summarized evidence on sea cucumber–derived peptides and polysaccharides, these findings strongly support the antioxidant potential of sea cucumber viscera. Nevertheless, further validation in animal models and exploration of clinical applicability are warranted to fully establish its efficacy and translational prospects.

### 4.5. Anti-Aging Effect

Aging is characterized by the progressive accumulation of cellular damage and functional decline in organisms, ultimately leading to diminished adaptability, increased disease susceptibility, and elevated mortality risk [102]. Recently, López-Otín and colleagues identified twelve indicators of aging, including mutations in the genome telomere attrition, epigenetic changes, decreased proteostasis, disrupted nutrient sensing, dysfunctional mitochondria, cellular degeneration, stem cell depletion, altered intercellular relationships, continuing inflammation, and imbalances in bacteria [103]. Age-associated microbial dysbiosis may lead to improper aging and decreased longevity [104].

Although direct experimental evidence for the anti-aging effects of sea cucumber viscera is currently scarce, the viscera share a comparable bioactive profile with the body wall. Moreover, existing studies have identified that bioactive compounds isolated from the body wall exhibit certain anti-aging properties. This provides a rationale for the hypothesis that visceral components may likewise harbor intrinsic anti-aging potential. In a Drosophila model evaluating sea cucumber peptides (SCP) at varying concentrations, SCP supplementation prolonged the lifespan of both sexual flies, with a more pronounced effect in males. In the control group, the relative abundances of *Firmicutes* and *Actinobacteria* decreased, while those of *Proteobacteria* and *Bacteroidota* increased. SCP treatment reversed this trend, significantly elevating the levels of *Lactobacillus*, *Rhodococcus*, *norank_f__Propionibacteriaceae*, and *Propioniciclava*, while reducing the relative abundances of *Acetobacter*, *Achromobacter*, *Pedobacter*, and *norank_f__Saprospiraceae*. Further analysis suggested that SCPs may extend lifespan by modulating metabolites involved in amino acid, nucleotide, and carbohydrate metabolism, impacting pathways related to oxidative stress, energy metabolism, neurotransmission, and purine/pyrimidine homeostasis. Notably, while the 0.2% SCP group significantly increased the maximum lifespan of female flies, the 0.4%, 0.6%, and 0.8% SCP groups did not significantly affect maximum lifespan in either sex [104]. Taken together, these results suggest that sea cucumber viscera may possess anti-aging potential; however, this hypothesis warrants further empirical validation through direct experimentation on visceral components themselves.

In a parallel study using *Caenorhabditis elegans*, sea cucumber protein hydrolysates enhanced the organism’s antioxidant defense system, upregulating superoxide dismutase and catalase activities and reducing malondialdehyde levels. This treatment not only improved survival under oxidative stress but also decreased intracellular ROS accumulation. Given the established role of oxidative stress in aging, and considering that bioactive components from sea cucumber viscera can boost systemic antioxidant capacity via gut microbiota metabolism, it is plausible that viscera-derived compounds may possess anti-aging potential. Nevertheless, this hypothesis requires further experimental validation.

### 4.6. Other Potential Benefits

Beyond the health benefits previously discussed, active components derived from sea cucumber viscera also exhibit additional physiological effects, including anti-fatigue properties and uric acid reduction.

Research indicates that glycolysis and the tricarboxylic acid (TCA) cycle are central metabolic pathways crucial for energy production [105]. Intake of sea cucumber viscera significantly increased the abundance of key bacterial genera involved in these pathways, such as *Dubosiella*, *Ventrimonas*, and *Bifidobacterium* within the glycolytic pathway. Similarly, *Bifidobacterium*, *Dubosiella*, *Paramuribaculum*, *Duncaniella*, and *Ventrimonas* were notably enriched in relation to the TCA cycle [55]. This dual modulation of microbial populations associated with core energy metabolism pathways is implicated in the observed anti-fatigue effects.

Hyperuricemia, a chronic metabolic disorder primarily characterized by increased serum uric acid levels, results from dysregulated purine metabolism. A comparative study evaluating the effects of hydrolysates from Japanese sea cucumber and white sea cucumber demonstrated that both treatments alleviated diet-induced hyperuricemia and renal inflammation in ICR mice. The underlying mechanism involves the inhibition of uric acid biosynthesis and the promotion of its excretion, mediated through modulation of the gut microbiota. Specifically, supplementation with either hydrolysate upregulated the abundance of beneficial *lactobacilli* and SCFA-producing bacteria while reducing opportunistic pathogens [106]. *Lactobacilli* contribute to uric acid reduction through a dual mechanism. The direct enzymatic catabolism involves the production of uricase, allantoinase, and allantoicase, which sequentially degrade uric acid to 5-hydroxyisourate, allantoin, and urea. Concurrently, they attenuate hyperuricemia indirectly by reducing intestinal purine absorption, thereby lowering serum uric acid levels [107].

Furthermore, sea cucumber viscera contain various bioactive polypeptides. Studies indicate that oligopeptides from *Apostichopus japonicus* can modulate uric acid metabolism by reshaping the gut microbiota, suppressing the NLRP3 inflammasome and the NF-κB pathway, and restoring the gut barrier, collectively contributing to the alleviation of hyperuricemia [108].

## 5. Research Challenges and Future Prospects

**Figure 4:**
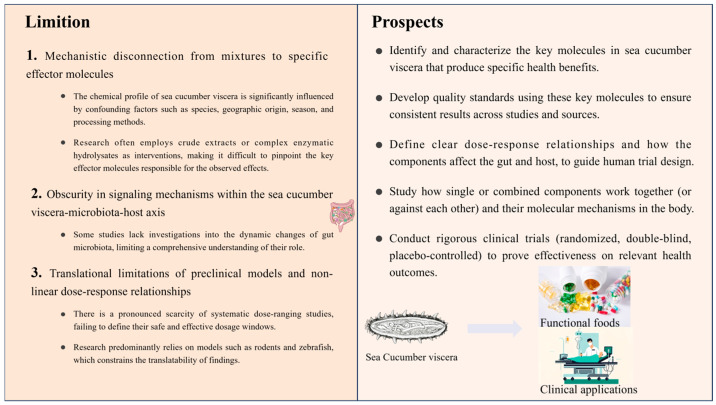
Current challenges and future research directions for the development of sea cucumber viscera as gut microbiota modulators.

Recently, the expansion of sea cucumber aquaculture has drawn attention to the comprehensive utilization of processing by-products, particularly sea cucumber viscera. The viscera contain various components such as proteins, polysaccharides, and saponins, which exhibit a range of biological capacities including antioxidant, antidiabetic, ACE-inhibitory, immunomodulatory, anticancer, anti-fatigue, anti-aging, neuroprotective, and trace mineral-chelating effects [92,109]. Although accumulating evidence suggests that sea cucumber viscera possess bioactive components with the potential to improve wellbeing by modulating the intestinal microbiome, their translation into functional foods and clinical applications still faces multiple challenges (Figure 4).

Sea cucumbers have specific living habits that make them susceptible to heavy metal contamination from sediments, leading to residual heavy metal accumulation. Studies indicate that the viscera (including the digestive tract, respiratory tree, and excretory organs) of sea cucumbers exhibit a significantly higher enrichment capacity for heavy metals such as Pb, Cd, As, and Cr compared to the body wall [110]. Exposure experiments further confirm that the accumulation level and rate of lead in the visceral mass are greater than those in the body wall [111]. This highlights that the removal of heavy metals from sea cucumber viscera represents the primary threshold for their safe use as food. Notably, the enzyme-chemical method is recognized as an effective approach for efficiently eliminating heavy metals. Researchers have identified optimal removal conditions as follows: hydrolysis with 0.10% (*w*/*w*) papain at 37 °C for 1 h, followed by immersion in 2.0% (*w*/*v*) food-grade citric acid for 48 h [112,113]. This method can achieve a heavy metal removal rate exceeding 90%. Future studies should further prioritize the safety assessment of heavy metals in viscera as a core aspect in the development of functional food products.

Current research predominantly focuses on the body wall, with significantly less attention dedicated to the viscera. Although compositionally similar, significant differences may exist in the structure and abundance of specific active substances—such as sulfated polysaccharides, saponins, and bioactive peptides—which directly influence their bioavailability and functional properties. Most existing studies employ crude extracts or complex enzymatic hydrolysates for intervention. While such approaches can reflect overall effects, they often fail to identify the key effector molecules driving specific physiological responses, let alone elucidate the precise interaction mechanisms between these components and gut microbes.

The health benefits of dietary components are chiefly regulated by the gut microbiota through a bidirectional interaction: dietary components shape the microbiota, which in turn metabolizes them into bioactive metabolites [114]. In fact, the majority of studies on sea cucumber viscera remain predominantly descriptive, limited to reporting in vitro bioactivities or terminal phenotypes in animal models. These investigations largely overlook the pivotal role of the gut microbiota as a central mediating hub. Consequently, it remains challenging to elucidate the mechanism of active components within the viscera are metabolically transformed by the microbiota, or how microbiota-derived signals (such as metabolites) subsequently coordinate their multi-target effects. It is noteworthy that sea cucumber viscera, particularly the intestines and gonads, contain carotenoids (astaxanthin and canthaxanthin) as well as taurine-rich peptides, which represent underexplored sources for multi-target interventions [115,116]. Further investigation is warranted to delineate their underlying mechanisms of action. Additionally, the profile and concentration of active components in sea cucumber viscera are significantly influenced by species, geographical origin, harvesting season, and farming methods, further highlighting the importance of isolating and identifying core functional constituents [38]. Beyond identifying the active components, researchers also need to determine the effective dosage. As Wang Qianqian pointed out, in a Drosophila lifespan model, only a 0.2% dosage of sea cucumber peptides showed a significant anti-aging effect, while higher doses (0.4–0.8%) did not produce the same outcome, suggesting that biological effects are not always linearly related to dosage [104]. Moreover, most current studies are mainly conducted on rodents (mice), zebrafish, Drosophila, and Caenorhabditis elegans. However, inherent differences exist between these models and humans in terms of gut microbiota composition, immune systems, and metabolic pathways [117]. Therefore, the gut microbiota regulatory patterns derived from animal experiments require further clinical verification.

In summary, although sea cucumber viscera hold great theoretical potential as a gut microbiota-targeting functional ingredient, their development faces considerable obstacles. Future research should focus on extracting effective single components from the viscera, studying their effective dosage and safety based on animal models, and exploring the interaction mechanisms of single or mixed components. Subsequently, clinical trials should be incorporated to comprehensively utilize sea cucumber viscera—a by-product of sea cucumber processing—and to develop corresponding functional foods or health products.

## 6. Conclusions

In summary, sea cucumber viscera represent a promising yet underutilized source of microbiota-modulating bioactive compounds. The evidence compiled in this review indicates that visceral-derived proteins, peptides, polysaccharides, and lipids exert significant prebiotic-like effects, promoting a shift in the composition and function of the gut microbiota toward a more beneficial state. This shift is characterized by an increased relative abundance of beneficial bacteria, a decreased relative abundance of pathogenic bacteria, and enhanced production of metabolites including SCFAs and indole derivatives. These microbial changes further contribute to host health benefits, such as improved immunological function and decreased harm caused by oxidation, improved glucolipid metabolism-related disorders, anti-aging and anti-fatigue effects, and lowered uric acid levels. However, current research still faces certain limitations. Future work should focus on: (1) the extraction and characterization of single bioactive components from the viscera; (2) the use of animal models to further explore the interaction mechanisms of both individual and combined components within the gut microecology; and (3) the implementation of phased human clinical studies to evaluate their efficacy, safety, and inter-individual variability. Addressing these limitations will help transform sea cucumber viscera from fishery waste into scientifically validated precision nutrition and functional foods, providing a sustainable strategy for improving gut health and preventing metabolic diseases.

## Figures and Tables

**Table 1:** The optimal extraction process of active peptides from sea cucumber viscera.

Sea Cucumber Varieties	Types of Enzymes	Enzyme Addition	Reaction Time	Temperature (°C)	pH	Material-To-Liquid Ratio (g/mL)	Results	Reference
Sea cucumber	neutral protease	3 μL/g	4 h	65	7.0	1:4	High polypeptide production at 303.0 mg/g, with modest peptide quantity of 775.40 mg/g.	[21]
*Stichopus japonicus*	Alcalase	1.9%	73 min	55	8.3	1:2	The ACE inhibition rate of the enzymatic hydrolysate reach 91.32%.	[22]
Sea cucumber	Alcalase	0.11 mkat/g	4 h	54	9.0	1:11.7	In the sea cucumber polypeptide solid obtained by freeze-drying, the polypeptide yield is 7.12%, and the protein content reaches 91.53%.	[23]
*Stichopus horrens*	compound protease	2.5%	10 h	52.66	6.71	1:2	more than 74% of the polypeptides have a molecular mass of less than 500 Da.	[24]
Sea cucumber	compound protease	2%	5 h	50	7.5	1:2	the ACE inhibition rate of sea cucumber visceral polypeptides reach 55.4%.	[25]
*Stichopus Japonicus*	trypsin	0.375 mkat/g	5 h	37	8.0	1:99	the content of polypeptides accounts for 52.68%, and the content of oligopeptides accounts for 47.25% in the enzymatic hydrolysate.	[16,20]
Sea cucumber	trypsin	2.56%	1 h	37	8.5	1:1.98	the ACE inhibitory rate of visceral polypeptide was 56.80%	[26]

**Table 2:** Regulatory effects of nutritional components in sea cucumber viscera on gut microbiota.

Nutritional Components	Model	Dose	Time	Increased Proportions of Bacteria	Decreased Proportions of Bacteria	Reference
Enzymatic hydrolysates of gonads	KM mice	0.6 g/kg, 1.2 g/kg.	4 weeks	*Bacteroidetes*, *Candidate_division_*TM7, norank_f_Family_XIII, *Alistipes*, norank_o_RF9, *Parabacteroides*, *Enterorhabdus*, *Rikenella*, *Odoribacter*, *Akkermansia*, norank_f_Erysipelotrichacea, *Lactobacillus*, norank_f__Prevotellaceae, Candidatus_Saccharimonas, *Roseburia*, *Ruminococcus*, *Anaerotruncus*.	*Firmicutes*, *Mucispirillum*, *Bilophila*, Candidatus_Arthromitus, norank_f_vadinBB60, RC9_gut_group, *Acetatifactor*, *Staphylococcus*, *Marvinbryantia*, norank_f_Defluviitaleaceae, unclassified_f_Clostridiales.	[44]
Enzymatic hydrolysates of gonads	Tilapia	500 mg/kg, 1000 mg/kg, 1500 mg/kg.	30 days	*Phylum Proteobacteria*, *Unclassified_k_norank*, *Phylum Verrucomicrobia*, *Phylum Chlamydiae*, *Saccharibacteria*, *Phylum Cyanobacteria*, *Phylum Firmicutes*, *Phylum Spirochaetae*, *Phylum Planctomycetes*, *Genus Aeromonas*, *Genus Enterobacter*, *Genus Rhizobium*, *Genus Deefgea*, *Genus Bosea*, *Genus Roseomonas*, *Genus Reyranella*, *Genus Ensifer*, *Genus Aeromonas*.	*Phylum Bacteroidetes*, *Phylum Fusobacteria*, *Phylum Actinobacteria*, *Genus Pseudomonas*, *Genus Cetobacterium*, *Genus Vogesella*.	[44]
Protein enzymatic hydrolysate from intestine-ovum	ICR mice	0.2 g/kg, 0.5 g/kg, 1.0 g/kg.	30 days	*Bacteroidota*, *Dubosiella*, *Bifidobacterium*, *Paramuribaculum*, *Duncaniella*, *Ventrimonas*	*Firmicutes*, *Patescibacteria, Romboutsia*	[55]
Tendon polysaccharide	mice	/	14 days	*Lactobacillus*, *Bacteroides*, *Akkermansia*.	*Prevotellacese_*UCG-001, *Proteus*, *Alistipes*.	[31]
Ovum hydrolysates	C57 mice	100 mg/kg, 200 mg/kg, 600 mg/kg.	14 days	*Bacteroidetes*, *Butyricimonas*, *Prevotella* sp., *Muribaculum*	*Oscillospira*, *Prevotella*,	[50]
Freeze-dried viscera powder	KM mice	200 mg/kg, 400 mg/kg, 800 mg/kg.	28 days	*Allobaculum*, *Turicibacter*, *Bifidobacterium*, *Akkermansia*	*Firmicutes*, *Clostridia*, *Bacilli*, *Lactobacillales*, *Clostridia-Clostridiales*, *Lactococcus*, *Clostridium ruminatium*	[56]

## Data Availability

Not applicable.

## Abbreviations

The following abbreviations are used in this manuscript:

SCFA	Short-chain fatty acids
ACE	Angiotensin converting enzyme
FCS	Fucosylated chondroitin sulfate
PUFA	Polyunsaturated fatty acids
FFAs	Free fatty acids
EPA	Eicosapentaenoic acid
TAGs	Triacylglycerols
DHA	Docosahexaenoic acid
DAGE	Diacylglycerol ethers
IA	Indole-3-acetaldehyde
IAA	Indole-3-acetic acid
IPA	Indole-3-propionic acid
IArA	Indole-3-acrylic acid
IAld	Indole-3-aldehyde
IET	Indole-3-ethanol
AhR	Aryl hydrocarbon receptor
MDA	Malondialdehyde
SCP	Sea cucumber peptides
TCA	Tricarboxylic acid
